# An Abdominal Aortic Pseudoaneurysm Revealing Behçet's Disease

**DOI:** 10.1155/2022/8286579

**Published:** 2022-01-27

**Authors:** Zineb Baba, Ahmed Mougui, Imane El Bouchti

**Affiliations:** Department of Rheumatology, Mohammed VI University Hospital, Marrakech, Morocco

## Abstract

Behçet's disease (BD) is a vasculitis with multisystemic manifestations. Articular involvement is frequent and benign whereas vascular complications are rare but serious and can form the onset of the disease. The assessment of the thickness of the common femoral vein wall is a new tool for the diagnosis of BD with good sensitivity and specificity. We report the case of a 52-year-old man diagnosed with BD revealed by an abdominal aortic pseudoaneurysm and a chronic monoarthritis. The first flare-up of BD can occur in men over 50 years of age. In a context of a multisystemic disease, lumbar pain should lead to the search of abdominal aortic aneurysm. The assessment of the thickness of the common femoral vein wall is accessible and should be used especially in challenging cases.

## 1. Introduction

Behçet's disease (BD) is a variable vessel vasculitis that appears in adults of 20 to 40 years of age, predominantly in Japan and the Mediterranean Rim [1]. Systemic manifestations include articular involvement which is usually benign and vascular injury which is rare but can lead to serious complications [2]. Diagnosis is based on the International Search Group (ISG) for BD or the International Criteria for BD (ICBD). The assessment of common femoral vein wall thickness is a recent tool that can help in the diagnosis of BD with a sensitivity and specificity around 80% [3]. We report the case of a 52-year-old man who was initially admitted for chronic monoarthritis and presented an abdominal aortic pseudoaneurysm leading to the diagnosis of BD.

## 2. Case Presentation

**Figure 1:**
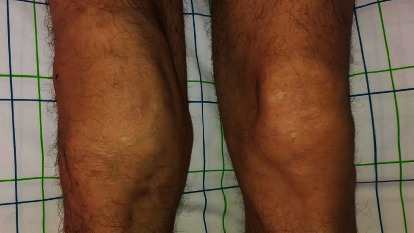
Arthritis of the right knee.

A 52-year-old man was admitted to our rheumatology department for chronic inflammatory arthralgia of the right knee that started swelling 4 months before (Figure 1). He also reported recent low back pain. His medical history only showed a 25 pack-year cigarette smoking. No history of hypertension, exposition to tuberculosis, or oro-genital ulcers was found.

Clinical examination of the patient found a right knee effusion with a positive patellar tap test and an abdominal pulsatile mass with a positive DeBakey sign. Examination of the spine and sacroiliac joints was normal.

**Figure 2:**
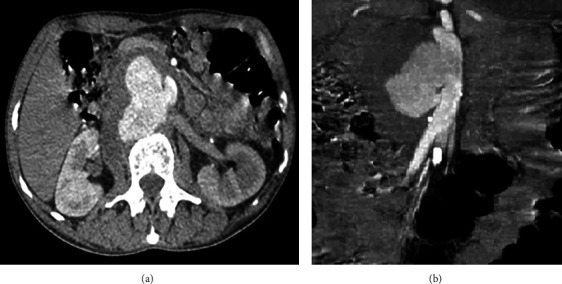
CT scan images showing the aortic pseudoaneurysm in an axial (a) and sagittal (b) view.

Abdominal ultrasonography and CT-angiography revealed an abdominal aortic saccular pseudoaneurysm measuring 58 × 70 × 91 mm which is partially thrombosed and associated with a periaortic hematoma and the absence of atherosclerotic calcification (Figure 2).

Aspiration of the knee joint brought an inflammatory fluid with a white blood cell count of 1760 cells/mm^3^ made of 70% of neutrophils. Gram stain and bacterial culture were negative. Blood tests showed an inflammatory anemia with 11 g/dl of hemoglobin, a white blood cell count of 6540 cells/mm^3^, and an erythrocyte sedimentation rate at 96 mm/h and a C-reactive protein at 67 mg/l. Sputum testing for tuberculosis was negative, including GeneXpert. Chest X-ray was normal. A synovial biopsy showed no sign of tuberculosis or malignancy. Radiography of the spine, sacroiliac joints, and knees showed no sign of spondyloarthritis. Syphilis testing in the blood and joint fluid was negative. Serology testing for hepatitis B virus, hepatitis C virus, and HIV was negative. Rheumatoid factor and anti-CCP, antinuclear, and antiphospholipid antibodies were negative. The pathergy test was negative.

**Figure 3:**
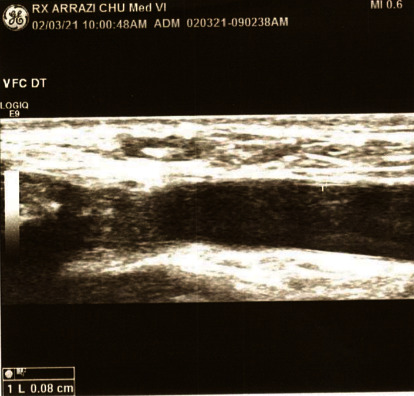
Image showing sonographic measurement of the thickness of the right common femoral vein wall at 0.8 mm.

Given the vascular and articular signs, the diagnosis of BD was suspected and the measurement of the thickness of the common femoral vein wall was made by Doppler ultrasonography, which was estimated 0.8 mm right and 0.7 mm left (Figure 3). These results were highly suggestive of BD [3].

**Figure 4:**
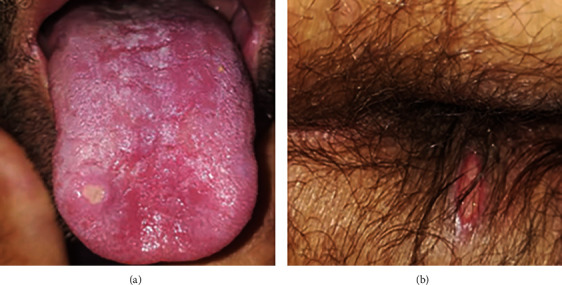
Oral (a) and genital (b) aphthosis.

During his stay at the hospital, the patient developed genital and oral ulcers (Figure 4) consistent with BD. At this stage, our patient was diagnosed with BD according to the International Search Group (ISG) for BD with a score of 3 and to the International Criteria for BD (ICBD) with a score of 4.

The patient received three successive intravenous pulses of methylprednisolone dosed at 1 g/day and was put on cyclophosphamide 1 g every 4 weeks and colchicine 1 mg per day.

The swelling of the knee diminished, and postbolus C-reactive protein was at 7 mg/l.

The patient was then referred to the cardiovascular surgery department for specialized management.

## 3. Discussion

Behçet's disease (BD) is a chronic inflammatory disease first described by Hulusi Behçet in 1937. It is characterized by an oral or oro-genital aphthosis associated with various systemic manifestations that can be mucocutaneous, ocular, articular, neurological, vascular, or gastrointestinal.

The anatomical substratum of BD is vasculitis. It is mainly seen in Japan and the Mediterranean Rim (the Silk Road). The onset generally occurs between 20 and 40 years of age. Juvenile BD is rare, and onset after 50 years of age, as for our patient, is exceptional. On a global scale, BD tends to affect men and women equally. However, men seem to be more affected than women in Mediterranean countries whereas female predominance is notable in Asian countries [1].

The primary mucocutaneous lesion is recurrent oral aphthosis. Genital aphthosis occurs in 60% to 80% of patients. Perineal aphthosis is less common in BD [2].

Articular involvement is most commonly benign, contrary to the ocular, vascular, and neurological complications which may have poor prognosis. It is often early and can inaugurate the disease. It includes arthralgia or oligoarthritis of the lower limbs. Monoarthritis is rare. Articular complications in BD are exceptionally destructive. Axial involvement is not rare in Behçet's disease. It includes spine pain and sacroiliitis. An association between BD and spondyloarthritis is also possible (2%) [4, 5]. In the context of aneurysm, refractory back pain should also trigger a search for vessel dissection or rupture; it can also be the result of vertebral erosion caused by the aortic aneurysm [6].

Ocular manifestations can be severe and include anterior or posterior uveitis and retinal vasculitis. Neurological manifestations can be parenchymal such as meningitis and meningoencephalitis or nonparenchymal linked to the impairment of cerebral vessels. Gastrointestinal manifestations are rare and consist mainly of diarrhea and abdominal pain [2].

Cardiovascular complications in BD occur in 7% to 38% of the cases [7], with a predilection for young male patients. Venous involvement is predominant and often happens during the first five years after the onset of BD. Venous, arterial, and cardiac impairments of BD often coexist [8, 9].

Cardiac manifestations can affect the pericardium, the myocardium, or the endocardium. Pericarditis (29%), which can be asymptomatic, is characterized by frequent recurrence. Intracardiac thrombosis occurs in 29% of the cases and affects mainly the right atrium and ventricle. They are frequently discovered along with thrombosis of other sites, particularly the inferior vena cava. 15% of cardiac complications are myocardial infarction, and 4% are aneurysms and pseudoaneurysms of the left ventricle [8, 10].

The most common venous complication is deep vein thrombosis, predominantly of the lower limbs. Deep vein thrombosis can also occur in the superior or inferior vena cava, cerebral veins or hepatic veins (Budd-Chiari syndrome). Pulmonary embolism and superficial vein thrombosis are also possible [8].

Arterial involvement occurs in 3-8% of patients [11]. This incidence may be underestimated according to a Japanese autopsy case study which found 34% of arterial impairment in patients with BD [12]. It is of late onset and includes pseudoaneurysms as the most frequent presentation, aneurysms, thrombosis, and stenosis. The abdominal aorta, the pulmonary artery, and arteries of the lower limbs are the predilection sites [8].

Histologically, the vasculitis of vasa vasorum leads to its obliteration and thus to the dysfunction of the nutrient supply to all three layers of the vessel wall. The inflammation process is responsible for abnormal localized dilatation of the vessel wall. The rupture of both intima and media layers causes leaking of the blood which becomes bounded by the outer vessel layer (adventitia) or perivascular tissue, resulting in the formation of a pseudoaneurysm, whereas in the true aneurysm, the media layer is weakened and the vessel is dilated within a soft spot in the vessel wall resulting in the formation of a true aneurysm, frequently saccular, where the blood is contained within all three layers of the vessel wall [9, 13].

Evaluation of common femoral vein wall thickness with Doppler ultrasound can indicate BD when higher than 0.48-0.49 mm, which was the case in our patient, with an approximate 80% sensitivity and specificity, regardless of vascular involvement [3].

Treatment of dermatological manifestations is based on topical corticosteroids and Amlexanox as an oral paste. Apremilast is a recent treatment that shows promise for oral ulcers [14]. Colchicine is used to prevent the recurrence of aphthosis. Colchicine is also used to treat acute arthritis whereas chronic and recurrent cases need the use of immunosuppressive drugs (azathioprine, interferon-alpha, and TNF-alpha inhibitors). More serious manifestations such as ocular, neurological, and vascular ones require the use of high-dose glucocorticoids and immunosuppressive drugs (azathioprine). High-dose glucocorticoids should never be used alone to treat anterior uveitis, and cyclosporine should be avoided in neuro-Behçet's disease [15].

Treatment of arterial involvement in BD consists in containing the acute phase by high-dose corticosteroids and immunosuppressive therapy (cyclophosphamide) [15], before endovascular or standard surgical intervention [16, 17], which are highly discouraged during the disease flare due to the high risk of complications. Endoscopy exposes the patient to the risk of aneurysm at the puncture site [15, 18] whereas open surgical repair is complex due to the extended healing duration and the difficulty to suture the friable vessel wall. Thus, surgeons prefer interventional endoscopy, although there is not enough evidence to state that it provides better prognosis for BD patients with pseudoaneurysm [9].

In vasculo-BD, arterial involvement is overall more severe than venous involvement. However, the own characteristics of each vascular complication can determine the prognosis. Thrombosis of the hepatic vein predicts poor prognosis during venous involvement with a 9 times higher mortality rate. 57% of deaths in arterial involvement are linked to aneurysm or pseudoaneurysm, mainly of the thoracic and abdominal aorta which can rupture or the pulmonary artery which can lead to massive hemoptysis. Septic and neoplasic side effects of immunosuppressant drugs are also major prognosis factors [8].

## 4. Conclusion

The onset of the first BD flare-up after the age of 50 is rare. Aortic pseudoaneurysm is infrequent, and its symptoms can be misleading. Prerupture syndrome of aortic aneurysm includes lumbar pain. The presence of monoarthritis associated with vascular involvement should alert practitioners to the diagnosis of BD. The evaluation of common femoral vein wall thickness is a helpful tool in the diagnosis of BD.

## Data Availability

The data used to support the findings of this study are included within the article.
